# How stable are the collagen and ferritin proteins for application in bioelectronics?

**DOI:** 10.1371/journal.pone.0246180

**Published:** 2021-01-29

**Authors:** Jayeeta Kolay, Sudipta Bera, Rupa Mukhopadhyay

**Affiliations:** School of Biological Sciences, Indian Association for the Cultivation of Science, Jadavpur, Kolkata, India; VIT University, INDIA

## Abstract

One major obstacle in development of biomolecular electronics is the loss of function of biomolecules upon their surface-integration and storage. Although a number of reports on solid-state electron transport capacity of proteins have been made, no study on whether their functional integrity is preserved upon surface-confinement and storage over a long period of time (few months) has been reported. We have investigated two specific cases—collagen and ferritin proteins, since these proteins exhibit considerable potential as bioelectronic materials as we reported earlier. Since one of the major factors for protein degradation is the proteolytic action of protease, such studies were made under the action of protease, which was either added deliberately or perceived to have entered in the reaction vial from ambient environment. Since no significant change in the structural characteristics of these proteins took place, as observed in the circular dichroism and UV-visible spectrophotometry experiments, and the electron transport capacity was largely retained even upon direct protease exposure as revealed from the current sensing atomic force spectroscopy experiments, we propose that stable films can be formed using the collagen and ferritin proteins. The observed protease-resistance and robust nature of these two proteins support their potential application in bioelectronics.

## Introduction

Integration of biomolecules, especially proteins, with electronic elements such as electrodes, chips, transistors etc. to form multifunctional electronic devices, is a crucial step in molecular bioelectronics [1]. Different types of proteins have been integrated so far in the structural framework of bio-electronic devices, for example, azurin and myoglobin in transistors [2–4], bacteriorhodopsin in bio-molecular transistors for data storage [5,6], genetically engineered α-haemolysin in bio-molecular circuits [7], photosystem I (PSI) in bio-hybrid solar cells [8], and cytochrome C and glucose oxidase in enzymatic biofuel cells [9–11]. Application of proteins in nanoscale bioelectronics is especially attractive since better device performance in terms of speed, functionality and reduced power consumption, as typically is the demand from the nanoscale electronic devices, can be achieved by using proteins, particularly the metalloproteins. This is because most of the metalloproteins naturally possess electron transfer capacity and unique nanoscale properties that are determined by their size, structure and electronic charge distribution. Metalloproteins can also be integrated onto standard substrates, be it the surface of a nanoscale object like nanoparticles or a larger micro-/millimetre length scale surface, without any significant loss of function, provided a proper surface anchoring procedure is followed. The metalloproteins have been found to be potential candidates for the construction of stable tunnel junction in the solid-state metal-insulator-metal configuration as they offer intrinsic capability of electron transfer over long distances (~10–20 Å) in a fast directional manner [2] and electron transport ability via the solid-state configuration [12].

In bioelectronic device fabrication, the stability of the biomaterial that is integrated in the device is one of the determining factors for the effective device utility. This is because retaining the biological activity of the surface-confined protein molecules is essential for successful development of bioelectronic devices. While a suitable immobilization method can prevent structural changes of the surface-anchored protein molecules, and therefore, loss of its biological function to a large extent, proteins can still be degraded under different conditions such as variation in temperature and the action of protease from the environmental sources upon storage of the protein-integrated component. So far, the chemisorbed azurin protein has been found to be structurally stable in air in solid-state conditions [13], hence considered to be useful for application in the solid-state devices. However, its stability upon storage for few months has not yet been reported. The supported purple membrane from *Halobacterium Halobium* could be stored for years without a significant loss of activity [14], although biological activity of membrane proteins like bacteriorhodopsin isolated from purple membrane in stable lipid bilayer on platinum substrate remained unaltered only for several weeks [15].

A few solid-state applications using ferritin and collagen proteins have been reported in recent times. For example, a ferritin-based protein bio-memory substrate, where ferritin molecules were immobilized onto graphene-modified glassy carbon electrode [16], and a ferritin-based bio-nanobattery [17], have been reported. Also, collagen-based films have been used in flexible implantable electronics [18]. However, no systematic investigation of the stability of the protein film, especially upon storage, has been reported so far. We have therefore tested whether the ferritin and collagen films can be stored for at least few months without change in electron transport characteristics so that these films can be put to use in bioelectronic device development.

Both the collagen and ferritin proteins are easily available because they are widely found in mammals, where ferritin is also found in lower organisms including the prokaryotes. Collagen is a primary structural protein and a main component of the extracellular matrix. It is composed of a triple helical polypeptide structure which consists of two identical α_1_ chains and one α_2_ chains [19]. Fibrillar collagen type I [20] provides the mechanical strength to skin, tendon, bone etc. Since collagen has a considerably long half-life of 117 years (cartilage collagen) or 15 years (skin collagen) [21], the shelf-life of collagen-based components is expected to be at least few months to few years. The ferritin protein consists of 24 subunits that lead to the formation of eight 3-fold channels and six 4-fold channels. It is stable up to ~80 °C temperature, and within a wide pH range of 4–9 [22]. It’s functional integrity is retained also when it is immobilized onto a solid substrate via the surface-exposed thiol groups [23–30]. The presence of the semiconducting iron core within ferritin makes it a potential candidate for application in bioelectronics [23–30]. Importantly, since the metal center of ferritin can be replaced with other biocompatible metals in a structurally conservative manner [31–34], its electron transport properties can be tuned [24,25].

Herein, we report the impact of the deliberately added bacterial protease and of the protease from environmental sources on the collagen and ferritin proteins, to test if protease can degrade and/or alter the protein structure to a detrimental level so as to interfere with the solid-state electron transport capacity of the respective protein films. In our work, we chose bacterial proteinase from *Bacillus Licheniformis*, which is an alkaline protease and is known to hydrolyze both native and denatured proteins, and a large number of different types of proteins [35,36]. Since the microbial alkaline proteases are one of the most widely used hydrolytic enzymes that are extensively used in industries such as tannery, dairy, food, pharmaceutical, silk, detergent, waste management etc. due to their general applicability, its use was deemed the most appropriate for the present study.

## Materials and methods

### Materials

All the buffer solutions were prepared using autoclaved filtered Milli-Q water (resistivity 18.2 MΩ·cm, Millipore). Disodium hydrogen phosphate (Na_2_HPO_4_), sodium dihydrogen phosphate (NaH_2_PO_4_), sodium chloride (NaCl), potassium dihydrogen phosphate (KH_2_PO_4_), potassium chloride (KCl) and tris(hydroxymethyl) aminomethane [(CH_2_OH)_3_CNH_2_] were purchased from Merck (purity ≥ 99%). Bovine achilles tendon (BAT) collagen, equine spleen holoferritin, bacterial proteinase (type XXIV) were procured from Sigma-Aldrich.

### Preparation of protein solutions

About 10 mg of bovine achilles tendon collagen was added in 2 mL of 0.01 M H_2_SO_4_ solution and stored overnight at 4 °C temperature with occasional mild shaking, followed by homogenization for 10 min at 0 °C. The solution was then diluted with 13 mM Na_2_HPO_4_, 2.5 mM NaH_2_PO_4_, 140 mM NaCl, pH 7.4 buffer to obtain 0.12 mg/mL collagen solution [37]. Ferritin from equine spleen (stock concentration: 125 mg/mL) was diluted with 0.3 mM tris, 0.15 M NaCl, pH 7.4 buffer to obtain 0.12 mg/mL ferritin solution.

### Preparation of protease-treated protein solutions

Although the main purpose of our work was to check whether the collagen and ferritin films remained stable in solid-state, however, as an additional confirmatory test for assessment of the stability of these proteins upon protease exposure, we treated the protein solutions with protease with the understanding that protease’s protein degrading action can be better performed in solution phase, rather than in solid-state. For preparation of the protease-treated protein solution by deliberate addition of protease, bacterial proteinase (8.58 μg/mL) solution in PBS buffer (pH 7.5) was added (1:200 v/v) to the collagen solution in 13 mM Na_2_HPO_4_, 2.5 mM NaH_2_PO_4_, 140 mM NaCl buffer (pH 7.4) or the ferritin solution in 0.3 mM tris, 150 mM NaCl buffer (pH 7.4) as the case may be. The collagen and the ferritin solutions were each equilibrated for 30 min at 37 °C temperature prior to addition of bacterial proteinase. The protease-treated protein solutions were incubated in dry bath overnight (around 15 h) at 37 °C temperature (i.e., the physiological temperature). As 37 °C is within the range of optimum temperature for protease activity, i.e., 30–40 °C [38], the protease activity at 37 °C should be considerably high. We kept the experimental temperature at 37 °C so that we could mimic the situation where such protein films are used in *in vivo* application (e.g., application of ferritin film) or next-to-skin application (e.g., application of collagen film). Once the collagen and the ferritin proteins were subjected to protease treatment, we think whatever protease-induced degradation could happen that took place within the incubation period of 15 h.

In order to prepare the environmental protease-exposed protein solutions, the collagen and the ferritin solutions taken in small hard glass tubes covered with perforated parafilm strip were kept in a humidity chamber at room temperature to minimize the rate of evaporation. The humidity chamber was kept slightly opened (about 0.5–1 mm since the chamber was kept covered with a lid although the chamber was not sealed with a sellotape), so that availability of environmental proteases inside the chamber would be the same as that outside of it. The temperature and relative humidity in the chamber should be 24±1 °C and about 75% for the saturated salt (NaCl)/water solution that we used. However, since the box was not tightly sealed, the humidity level could become less with time. For testing the effect of protease that is present in various environmental sources, the protein solution was stored for different time periods, starting from 1 week till 1 month. Since, protease entry could happen at any time point within this time intervals, we do not think protease half-life is an important factor in the present context.

### Preparation of heat-denatured protein solutions

For heat-induced denaturation, 0.12 mg/mL collagen solution in milli-q water (as per protocol in reference 39) was heated in dry bath (Genei dry bath, model no: DB 900, Volts/Hz: 230/50) at 70 °C temperature for 10 min [39]. Similarly, 0.12 mg/mL ferritin solution in 0.1 M NaCl solution was heated at 90 °C temperature for 10 min [22]. Here, we intentionally denatured the collagen and ferritin proteins by heat treatment so that we could know that if a protein is denatured then how the CD spectra will look like.

### Preparation of protein film on silicon surface

A heavily doped n-type (As) silicon(111) substrate having resistivity 0.0025–0.004 Ω.cm and of thickness 525 μm (University Wafer, USA) was cleaned by bath sonication in ethyl acetate/ acetone/ ethanol (5 min in each), followed by 10 min of acid piranha treatment (7:3 v/v of H_2_SO_4_:H_2_O_2_) at 80 °C temperature. Then the wafer was thoroughly rinsed with milli-q water and dipped in 2% HF solution for 2 min, followed by rinsing with milli-q water and then dipped in a base piranha solution (NH_4_OH:H_2_O_2_:H_2_O = 1:1:5) for 1 min at 70 °C temperature. The wafer was rinsed with milli-q water and dried under nitrogen gas. The procedures followed in our work are standard processes for substrate preparation for conductive measurements [40**]**. In a conductive measurement, the thickness of the silicon oxide on top of the silicon wafer is very crucial. In the present work, we chose heavily doped (As) n-type silicon <111> wafer having sufficient electronic conductivity to perform the CSAFS measurements. It is a well-known fact that upon storage in the normal ambient condition the silicon wafer gets coated with an insulating native silicon oxide layer which suppresses the electron conduction ability through it. Therefore, we needed this silicon oxide layer to be of the lowest possible thickness to retain enough electronic conductivity. At the same time, the top oxide layer plays a crucial role for protein immobilization process on this substrate. Hence, we followed the procedure for controlled growth of silicon oxide as also followed in our previous report [41] in order to satisfy both the requirements of the presence of the oxide layer for protein immobilization and to make it a thin layer so that the electron transport experiments can be performed. In this procedure, initially we removed the native oxide layer by HF etching to make the wafer oxide-free hydrophobic surface. Thereafter, we used the base piranha solution (relatively weak oxidising agent than acid piranha) for controlled growth of oxide layer (< 1 nm thick) [40] and for also removing the metallic contamination (e.g., iron) from the wafer surface [41**]**. The higher conductivity (lower resistance) of the silicon substrate with controlled oxide growth layer was indicated in kilo-ohm (few hundred kΩ) order resistance whereas the initial native-oxide-coated silicon exhibited higher mega-ohm (MΩ) level resistance.

The cleaned silicon wafers were incubated for 12 h in collagen solution prepared using 13 mM Na_2_HPO_4_, 2.5 mM NaH_2_PO_4_, 140 mM NaCl (pH 7.4) buffer, where either 0.1 mg/mL of freshly prepared collagen solution or the collagen solution exposed to ambient environment for 30 days, was taken. Then it was thoroughly rinsed with autoclaved milli-q water and dried under nitrogen gas. For preparation of ferritin film on silicon surface, the cleaned silicon wafer was incubated into 10% (v/v) of 3-APTMS in methanol for 3 h, followed by 3 min bath sonication in methanol, rinsed in milli-q water and dried under nitrogen gas [40]. Then the APTMS-treated Si wafer was incubated for 12 h in 0.044 mg/mL freshly prepared ferritin solution or the ferritin solution exposed to the ambient environment for 30 days in 0.3 mM tris, 0.15 M NaCl (pH 7.4) buffer. Then it was thoroughly rinsed using autoclaved milli-q water and dried under nitrogen gas. The protein film thickness was checked by nanoshaving experiments using contact mode AFM (see S1 Fig in Supporting information), and the film thickness was found to be 1.6–2.0 nm (in case of collagen) indicating the formation of a near-monolayer coverage, as the collagen fibril is 1.5 nm in diameter. The ferritin film thickness was found to be 9–9.5 nm indicating monolayer thickness, as the ferritin is known to be 12 nm in size.

### Characterization of protease-treated and heat-treated protein solutions by circular dichroism (CD) spectroscopy and UV-visible (UV-Vis) spectrophotometry

The protease-treated and the heat-treated protein solutions were characterized using CD spectroscopy and UV-Vis spectrophotometry. The CD spectra were collected within 190 to 400 nm wavelength range with 100 nm/min scanning speed using a quartz cell cuvette of 10 mm path length and a JASCO J-815 CD spectrometer at room temperature. All the spectra were the average of three consecutive scans for each sample. The CD spectra were subjected to minimal smoothing (using ‘Spectra Manager’ software provided with the JASCO J-815 CD spectrometer) so that no alteration of the signal and/or loss of signal occurred. The UV-Vis spectra were acquired using Varian Cary 50 Bio UV-Vis spectrophotometer and a 1 cm cuvette at room temperature.

### Current-sensing atomic force spectroscopy (CSAFS) data acquisition and analysis

The CSAFS experiments were performed in contact mode using an Asylum Research MFP3D AFM. AFM probes (MikroMasch, Estonia) with Ti/Pt-coated cantilevers having spring constant 2 N/m, tip radius ~35 nm, length 110 μm, and width 40 μm were used in all the experiments. All the CSAFS measurements were performed under ambient condition, i.e., immediate environment/condition surrounding the experimental set up, which is always a temperature- and humidity-controlled environment (24±1 °C, and ~40–50%, respectively) with normal laboratory illumination and gentle air circulation as allowed by air conditioners. The current-voltage (I-V) response curves were acquired between ±5 V sweep range for collagen and ±3 V for ferritin. This is because in case of ferritin, ± 3 volt was enough to penetrate inside the on-state regime, whereas in case of collagen, ±3 volt stood within the off-state regime, and therefore we had to set the experimental bias range at ±5 volt to move to the on-state regime of collagen. The observed difference in the range of the off-state regime could be linked to the protein structure since ferritin possesses a semiconducting iron core [27] but collagen is a metal-free wide band gap semiconductor [42]. The I-V traces were acquired at two different force loads, at low force 7–11 nN and at high force 71–75 nN. The I-V experiments were repeated for 8–10 different areas on a sample, and for 3–4 different samples prepared on different days.

### Statistical analysis

In case of each type of experiment, the inter-assay deviation was tested using different batches of sample on different days or by repeating the experiment using the same batch of sample on the same day. The current values for data variability (mean variation) analysis were acquired at -4.5 V and 4.5 V (for collagen) and at -2.8 V and 2.8 V (for ferritin) from about 100 curves in each case. In order to test if the protein films were degraded due to storage over different time periods or different storage conditions, the significant difference in the current values was measured for the cases (i) freshly prepared protein film and film stored for 1 month, (ii) freshly prepared protein film and film stored for 3 months, and (iii) freshly prepared protein film and protease-exposed protein film. The statistical analysis was performed using Prism 8 software (GraphPad Software, Inc., San Diego, California). A p value of <0.05 was considered statistically significant.

## Results and discussion

In the present study, the action of protease on structural and functional integrity of collagen and ferritin proteins has been tested using CD spectroscopy, UV-Vis spectrophotometry and CSAFS. We have considered two types of proteases, i.e., bacterial proteinase that is deliberately added to the collagen/ferritin solution, and the protease that could be present in the immediate environment of the experimental setup. The far UV-CD spectra acquired in the region 190 to 260 nm were used to find out if any change in the secondary structure of collagen and ferritin occurred due to the action of protease. The UV-Vis spectra were used to test if the electronic structure of collagen and ferritin proteins were intact upon exposure to protease. The CSAFS was employed to check if the function of the two proteins in terms of their electron transport capacity remained unaltered, before and after protease exposure.

**Fig 1:**
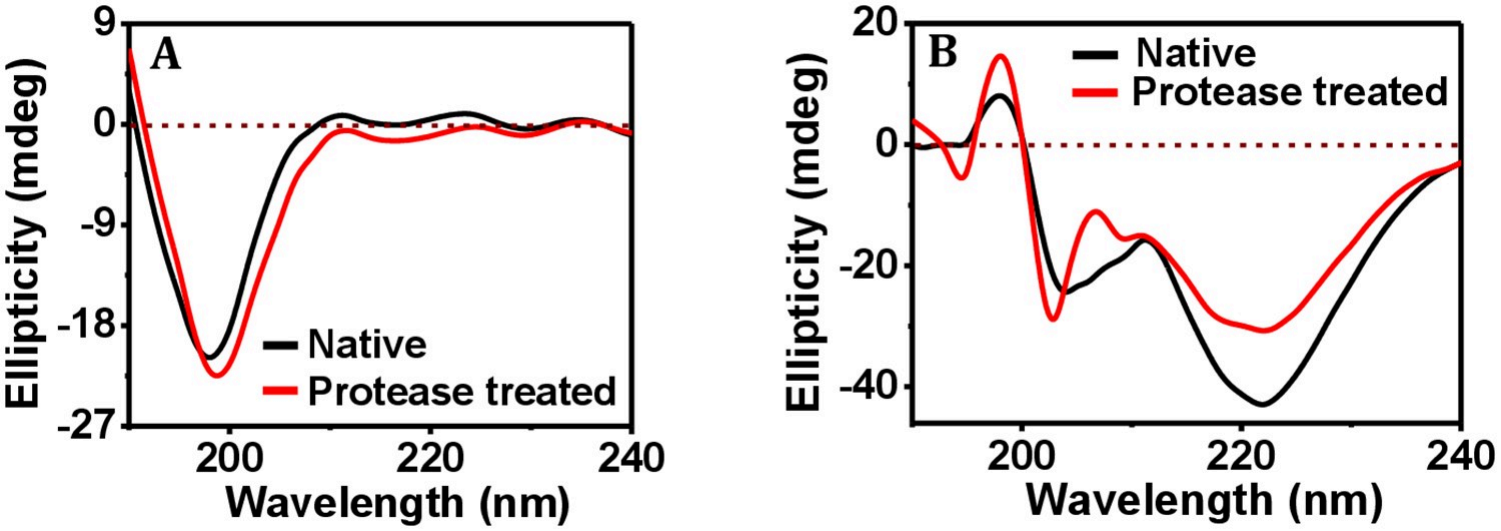
Effect of bacterial protease addition on the secondary structure of proteins. Representative far UV-CD spectra of (A) collagen, and (B) ferritin, before and after addition of bacterial protease.

In Fig 1A, the CD spectrum of collagen (black curve) that exhibits a prominent band at 197.5 nm wavelength with -21.6 ± 4 mdeg ellipticity is presented. The observed peak position is in accordance with the reported data [43]. However, the positive ellipticity band that is typical for triple helix collagen was very weak in our case. It could be due to an effect of low collagen concentration as reported earlier [44,45], where the ratio of negative CD band over the positive CD band for native collagen was found to be around 10:1. In our case, native collagen of 0.12 mg/mL concentration showed the negative ellipticity band at the maximum of -26 mdeg ellipticity, meaning that the positive CD band ellipticity value should not have crossed 2.6 mdeg. Upon protease treatment, the band at 197.5 nm shifted only slightly to 198.1 nm wavelength with -23.5 ± 6 mdeg ellipticity (Fig 1A, red curve). Since the 197 nm band weakens as a result of thermal denaturation [45,46], which has been verified by us as we observed a low ellipticity value (-8.47 mdeg) for the heat-treated collagen (S2A Fig), and also that gelatin, i.e., the denatured form of collagen, does not exhibit any characteristic CD signal [47], we conclude that no significant change in the secondary structure of collagen occurred and the protein did not denature due to protease exposure in the present case.

In case of the ferritin protein, the CD spectrum showed bands at 222 nm (ellipticity: -42.9 ± 5.7 mdeg), 203.9 nm (ellipticity: -24.3 ± 11 mdeg) and 197.9 nm (ellipticity: 8.2 ± 3 mdeg) (Fig 1B, black curve). The shape of the curve and magnitude of these far UV-CD bands suggest that ferritin protein has a substantial helical content and the polypeptide chains exist in an ordered structure [48,49]. For the protease-treated holoferritin, the bands observed at 222.1 nm, 202.8 nm and 198 nm (Fig 1B, red curve) indicate minimal shift in the band positions, although the ellipticity values, i.e., -30.7 ± 0.8 mdeg, -28.7 ± 2 mdeg and 14.6 ± 9 mdeg for the bands at 222.1 nm, 202.8 nm and 198 nm, respectively, are significantly altered. In order to establish that no feature in the observed ferritin spectrum appeared due to the added protease, a control CD experiment was performed using a protease-only solution (S2B Fig). The protease concentration of 8.58 μg/mL did not give rise to any CD response. Since protease was added to the ferritin solution at this concentration for the protease-treatment experiment, we conclude that the presence of protease did not result in any protease-specific feature in the CD signal of ferritin. Because a clear reduction took place in the ellipticity value of the 222 nm band, which is the major band that characterises the structure of ferritin [50,51], we conclude that treatment of ferritin with bacterial protease resulted in the diminished α-helix content of ferritin, which is not identical to the denaturation of protein. This is because if the protein was denatured then the α-helix would have been destroyed completely and the corresponding band for α-helix could not be seen. In the present case of the protease-treated ferritin, only the ellipticity value for the characteristic α-helix band was reduced. If the protein was denatured upon protease treatment then the band for α-helix would have disappeared. In the control experiment, where the ferritin solution was heated for denaturation, as performed for investigating changes in the CD spectrum that could take place upon denaturation of ferritin, the CD spectrum of the heat-treated ferritin was found to be quite different from that of native ferritin. Although it showed a strong negative peak at 222.5 nm wavelength, the two other peaks at 214 nm and 201.7 nm (S2C Fig) neither matched in peak position nor in the ellipticity values with the peaks observed for native ferritin (Fig 1B). As the CD spectra of the heat-denatured protein (S2C Fig) was largely different from the native one (Fig 1B), whereas the spectra for the protease-treated protein (Fig 1B) was not so different from the native one (Fig 1B), we concluded that the protease-treated ferritin protein was not denatured. In case of denaturation of ferritin using 7 M guanidinium chloride solution (pH 7.5), which is a strong protein denaturant, the negative ellipticity bands of ferritin are found to be almost absent [48]. The substantial changes in the peak position and in the ellipticity values as observed in cases of heat treatment and guanidinium chloride treatment, and an absence of any significant change in the CD spectrum in the case of protease-treated ferritin, indicate that protease exposure did not affect the ferritin protein to any significant extent.

**Fig 2:**
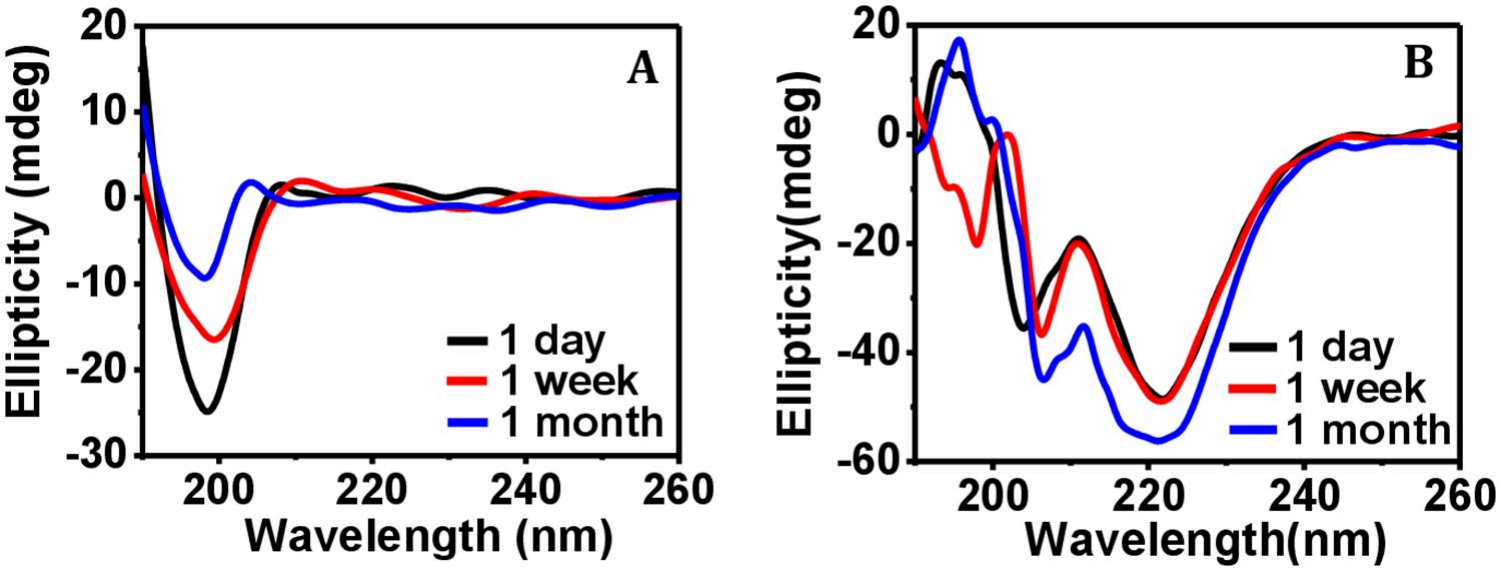
Action of environmental protease on the secondary structure of proteins. Representative far UV-CD spectra of (A) collagen, and (B) ferritin upon exposure to environmental protease for different time intervals.

After assessment of the effect of deliberate addition of bacterial protease to both collagen and ferritin proteins, we checked if any effect of the protease present in ambient environment, for example, allergen-derived proteases and microbial proteases [52], could be detrimental if the protein solutions were kept environment-exposed for a long time. The action of the environmental protease on protein solutions was measured at different time intervals (1 day, 1 week, and 1 month) using CD spectroscopy. From the far UV-CD spectra of collagen, the negative ellipticity bands were observed at 198.1 nm, 198.7 nm and 196.5 nm for day 1, day 7 and day 30, respectively (Fig 2A). Because in all the cases, the negative ellipticity band of collagen (at -20.1 ± 9.5 mdeg) is found to be mostly close to the band observed for native collagen, we conclude that the action of environmental proteases on collagen was not severe.

The observation of small variation of the negative CD band position (Fig 2A) could not be attributed to the denaturing effect of protease. This is because it has been reported earlier that intensity and peak position in the UV-CD spectra of collagen can be dependent on collagen concentration [44]. It was also mentioned that such an observation does not imply either partial or entire denaturation of collagen at all [44]. That is because gelatin, which is the denatured form of collagen, does not exhibit any characteristic CD signal [47]. Therefore, we conclude that the denaturing effect of environmental protease on collagen is not present in our case, since if it was, no characteristic CD signal could be observed. However, some degree of cleavage was reported for collagen by SDS-PAGE electrophoresis experiments [53] and therefore the related contribution may be present in the observed CD spectra (Fig 2A) for the collagen protein.

No significant effect of the environmental proteases on ferritin protein could be detected in the CD spectra (Fig 2B). The negative ellipticity band (ellipticity value -50.7 ± 5 mdeg) at about 221 nm was found to be common for all the three cases (1^st^, 7^th^ and 30^th^ day), whereas the other band positions (at about 204 nm, ellipticity value -45 ± 9 mdeg) varied to some extent (within 2 to 3 nm). Overall, the CD spectra of the ferritin protein remained almost the same even after keeping the solution exposed in ambient environment for 1 month, indicating structural stability of the environment-exposed ferritin protein over the observed time period. Although in case of bacterial protease treatment, the ellipticity value for the 222 nm band was clearly reduced (Fig 1B), there was little change in case of environmental influence (Fig 2B), meaning that the α-helix content of ferritin was not altered to any significant extent after environmental exposure. Importantly, no cleavage of the ferritin protein could be observed in SDS-PAGE electrophoresis experiment [54]. Since our purpose was to test the storage capacity of the protein films in normal indoor environment, the minimal change in the ferritin CD spectra upon environmental exposure is of greater relevance than the effect of deliberately added bacterial protease. In general, the environmental protease should not be a concern as in our case since its amount must be too small in the normal laboratory condition.

**Fig 3:**
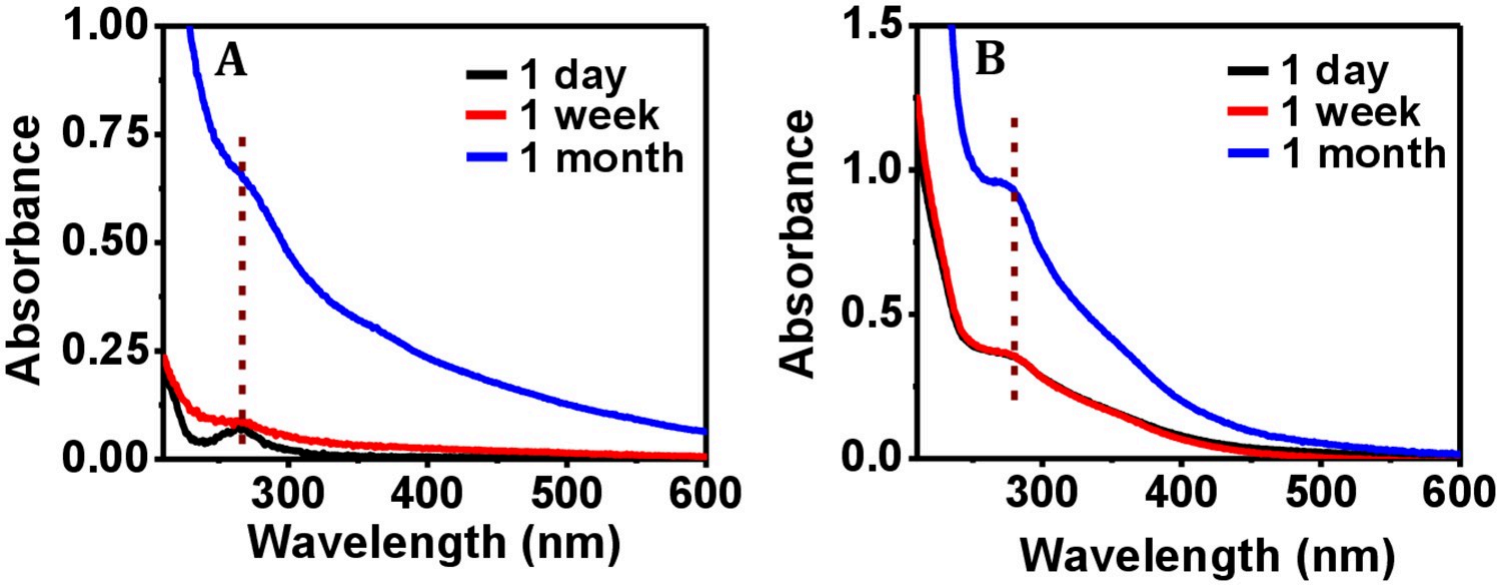
Effect of environmental protease on the UV-visible spectrophotometric data of proteins. Representative UV-Vis spectra of (A) collagen, and (B) ferritin upon exposure to environmental protease for different time intervals. The peak positions are shown by the segmented lines.

Apart from CD spectroscopy, the effect of protease on the collagen and ferritin proteins was studied using UV-Vis spectrophotometry. Upon exposure to environmental protease for different time intervals, the peak positions for collagen and ferritin did not alter and remained fixed at 265.5 nm (Fig 3A) and at 280 nm (Fig 3B), respectively. The observed increase in the absorbance value with increasing time intervals that did not happen before about 1 week (S3 Fig) could be due to solvent evaporation and an associated increase in the protein concentration.

**Fig 4:**
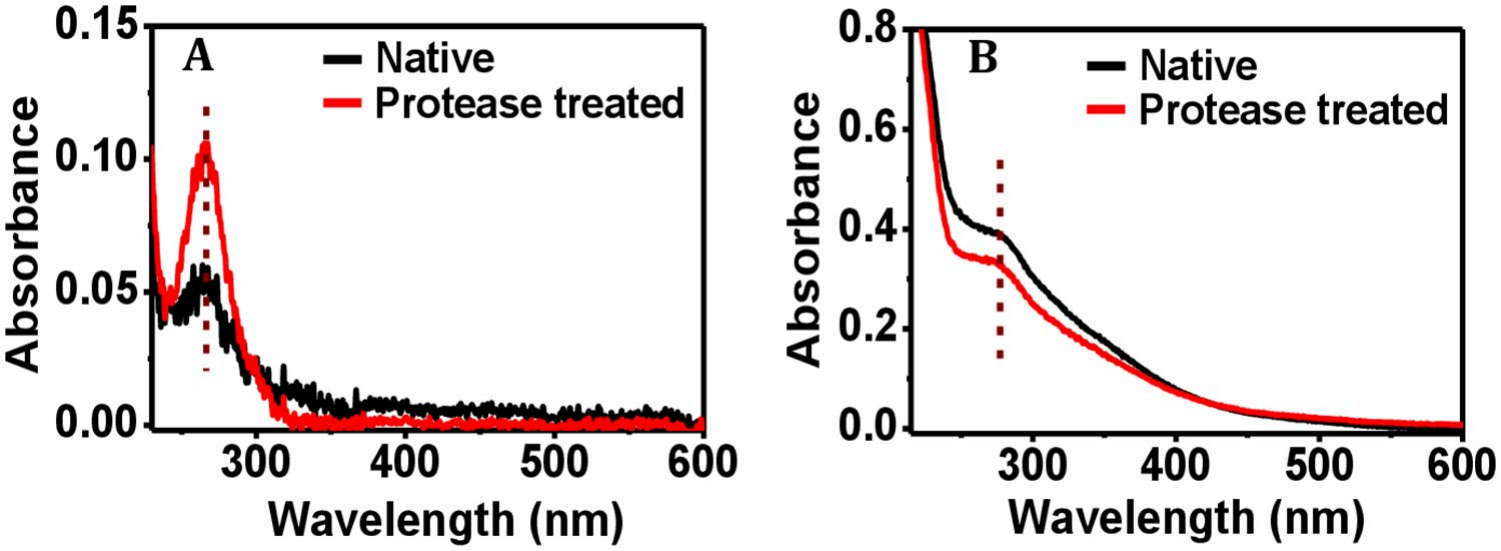
Effect of bacterial protease on the UV-visible spectrophotometric data of proteins. Representative UV-Vis spectra of (A) collagen, and (B) ferritin, before and after addition of bacterial protease. The peak positions are shown by the segmented lines.

Upon deliberate addition of protease to collagen protein, the absorbance value increased, although the peak position remained fixed at 265.5 nm (Fig 4A). The increase in the absorbance value could be due to the added protease, since protease of the same concentration as the added one gives rise to a distinct peak at nearly the same wavelength (S4 Fig). It is clear in Fig 4A and S4 Fig (the protease-only case) that native collagen and the added protease have their characteristic UV-absorbance at 265.5 nm with ~0.06 and ~0.03 absorbance, respectively. So the bacterial protease-treated protein solution showed the combined UV absorbance of ~0.1. In case of ferritin, the peak position at 280 nm did not change with addition of protease, while a decrease in the absorbance value was observed (Fig 4B). In this case, the absorbance at 280 nm is found to be ~0.4. Since 280 nm is not the peak position for protease (see S4 Fig), therefore the protease absorbance was not effectively added to the net absorbance value. Rather, due to dilution of the ferritin solution after addition of protease solution, the net UV-absorbance at 280 nm was reduced to ~0.35.

In order to check if the denatured collagen and ferritin proteins gave rise to a UV-Vis response at all, we performed a control experiment using heat-denatured collagen and ferritin solutions. The peak character was lost and a broader band appeared, and an increase in the absorbance value was observed in case of both the proteins at the same wavelength as that for the native proteins (S5 Fig). The denaturation-induced increase in the absorbance value could be due to solvent exposure of the amino acid residues like tyrosine and tryptophan that were exposed to the protein surface upon denaturation [55]. It has been observed earlier that after adding denaturants to the protein solution, the absorbance of tyrosine and tryptophan increases without any structural transitions [56]. Because the peak character of the protein samples was not lost upon protease treatment (Fig 4), it can be concluded that protease treatment of the collagen and ferritin proteins minimally affected the protein structures.

**Fig 5:**
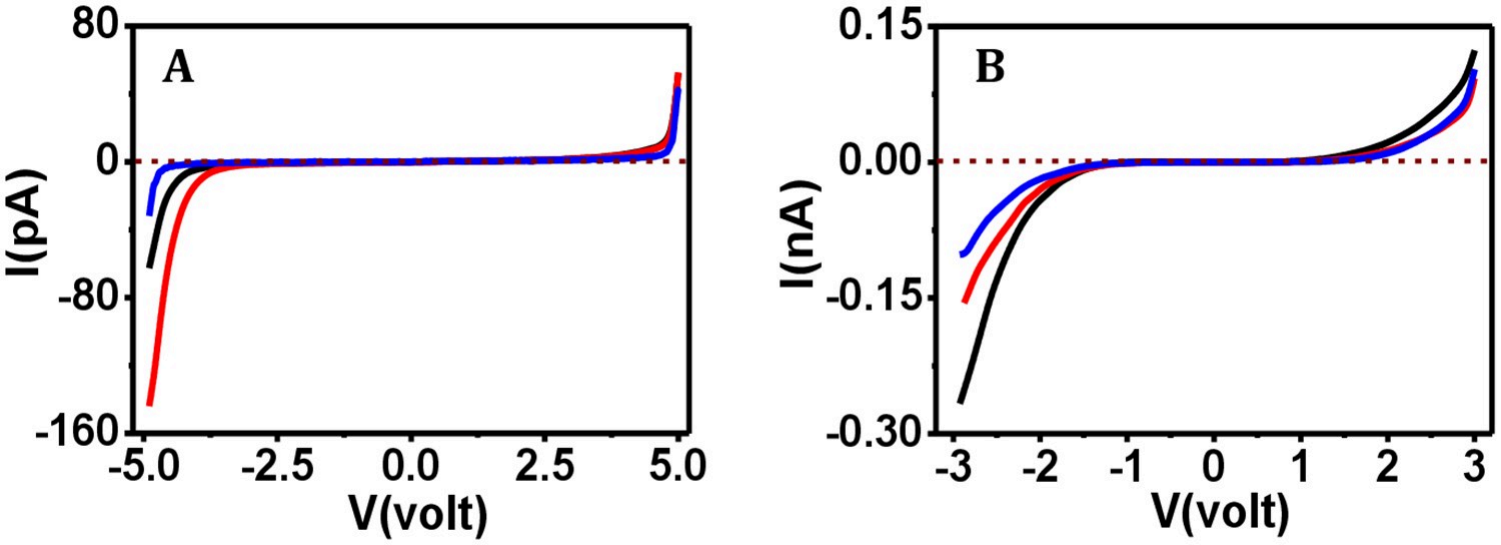
Electron transport capacity of stored protein films measured at different time intervals. The I-V curves for (A) collagen and (B) ferritin films that are freshly prepared (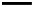), and films stored for one month (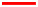), and three months (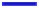), at 71–75 nN force applied.

Once the structural integrity of the protease-exposed proteins was found to remain mostly unaffected, we set forth for testing the electron transport capacity of both the proteins upon storage for a few weeks to a few months. Freshly prepared protein films were kept in covered petri dishes (but not sealed with parafilm strip) and stored at room temperature and under humidity level of 40–50%. We assumed that over such storage of the protein film sample there could be an exposure to protease from the airborne microbes by chance. We performed this experiment in order to mimic the situation for a device being stored in factory or in a laboratory after delivery for a considerably long time and assess the fate of its electron transport capacity. In reality, since the storage of electronic devices and components can be done by vacuum sealing, our setup presents an even more drastic case for testing. The electron transport capacity of the protein films was tested at different time intervals by current-voltage (I-V) measurements using CSAFS approach. The I-V curves acquired from the freshly prepared collagen film could sense current in the pA level (Fig 5A, black curve). When measured at the sweep voltages 4.5 V and -4.5 V, the respective current values were 7.8 pA and -16.5 pA under the force load of 71–75 nN. After storing this collagen film for one month, the current values were found to be 7.1 pA and—54.4 pA (Fig 5A, red curve), and after three months 3.1 pA and -2.6 pA (Fig 5A, blue curve) at +4.5 V and -4.5 V sweep voltages, respectively, under high force (71–75 nN) operation. The overall current was observed to be in the picoampere level whatever be the voltage or the time interval. In our previous work on collagen [42] too, the current from the collagen film did not follow a fixed order for varying conditions, for example, with increasing force the current values increased in the negative voltage region, whereas at the positive voltage, the current values sensed for the increasing force loads did not follow the same order. We suggest that the main concern in the present work should be to check whether the stored collagen gives rise to a current value as per its natural capacity, i.e., at the picoampere level. In case of ferritin, the freshly prepared film sensed current in the nA level, i.e., 0.08 nA, -0.23 nA at high force (71–75 nN) at sweep voltages +2.8 V and -2.8 V, respectively (Fig 5B, black curve).

Whereas, when this ferritin film was stored for one month the current values of 0.05 nA, -0.14 nA (Fig 5B, red curve), and after three months of storage the current values of 0.05 nA, -0.09 nA (Fig 5B, blue curve) were recorded +2.8 V and -2.8 V sweep voltages, respectively, under high force (71–75 nN) operation. All the on-states and the off-states for both collagen and ferritin proteins are shown in S1 Table and S6 Fig (Supporting information). In spite of some variations observed, the overall current was at the nA level, which is reflective of the fact that the natural electron capacity of ferritin was retained. These observations clearly indicate that both the proteins remained functionally active at room temperature and under humidity level of 40–50% even after few months of storage. Remarkably, the electron transport capacity was largely retained in both the proteins, even after nine months’ storage (S7 Fig), except for the observation of a widened band gap in case of collagen. Beyond nine months however the characteristic I-V response could be obtained only for the ferritin film, but not for the collagen film. In order to observe the characteristic I-V response in case of the collagen film, we had to increase the sweep voltage range from ±5 V to ±9 V. In case of the ferritin film also, the sweep voltage range had to be increased from ±3 V to ±6 V or more beyond the time period of 12 months. Therefore, we did not try testing over longer time periods.

**Fig 6:**
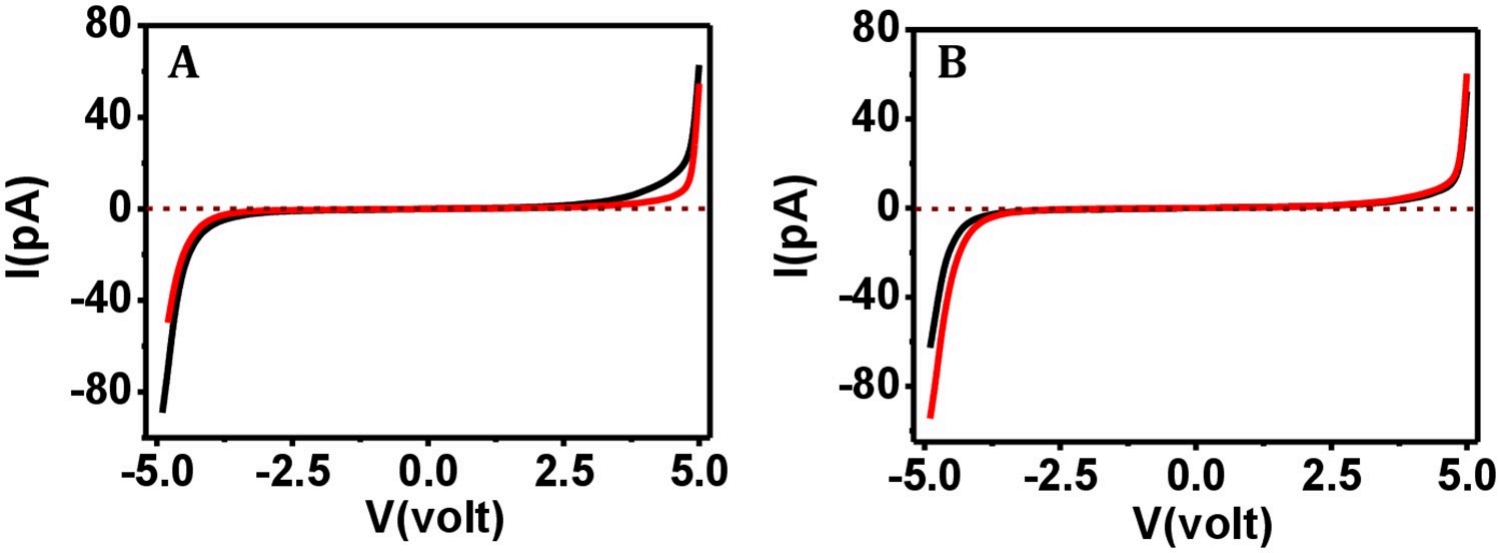
I-V data comparison for collagen where films prepared from fresh vs. environment exposed solutions. The I-V curves for freshly prepared collagen film (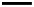), and film prepared using the solution that is kept environmentally exposed for 30 days (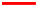) at (A) 7–11 nN and (B) 71–75 nN applied force.

We further tested the electron transport capacity of the collagen and ferritin proteins using protein solutions that have been exposed in the ambient environment for 30 days. These stored protein solutions were used for preparing the films on silicon surface and then I-V curves were acquired using CSAFS. We found that the freshly prepared collagen film could sense current in pA level (Fig 6A and 6B; black curves), where the collagen film prepared using stored protein solution could also sense current at pA level (Fig 6A and 6B; red curves), at both the low (7–11 nN) and high force (71–75 nN) regimes.

**Fig 7:**
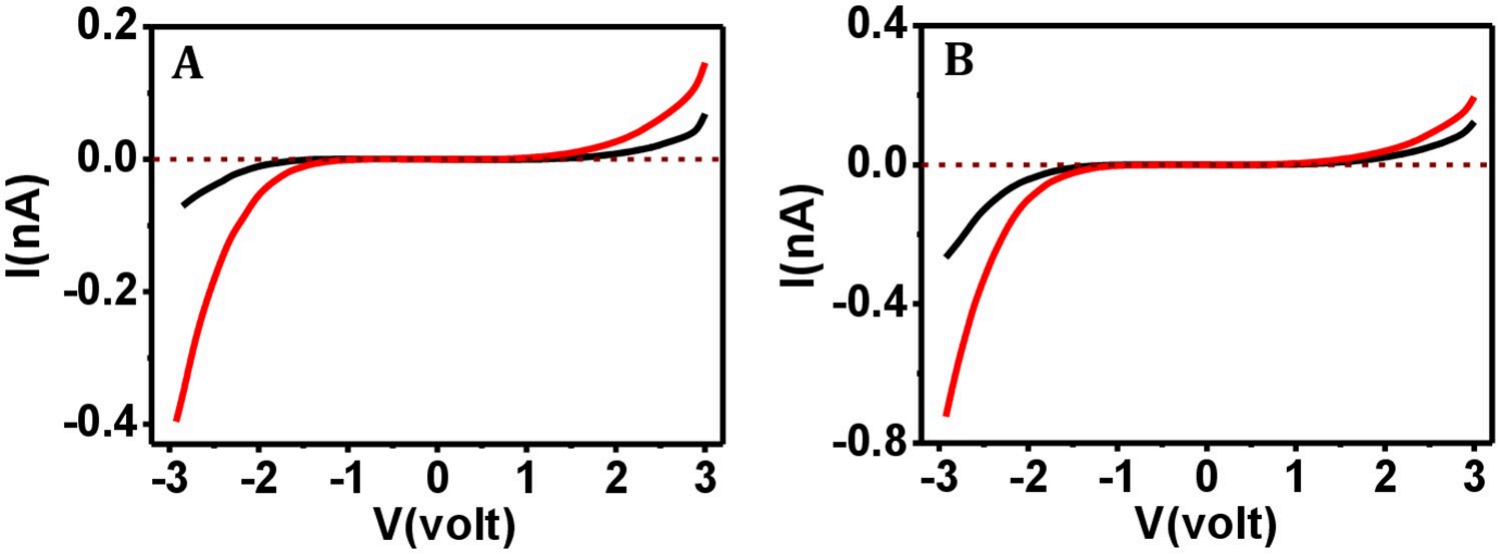
I-V data comparison for ferritin where films prepared from fresh vs. environment exposed solutions. The I-V curves for freshly prepared ferritin film (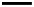), and film prepared using the solution that is kept environmentally exposed for 30 days (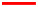) at (A) 7–11 nN and (B) 71–75 nN applied force.

The freshly prepared ferritin film was found to sense current at the nA level (Fig 7A and 7B; black curve), where the film prepared using the stored ferritin solution could also detect current at the nA level under both low (7–11 nN) and high force (71–75 nN) operation (Fig 7A and 7B; red curve). We therefore conclude that both collagen and ferritin samples remained functionally unharmed under environmental exposure.

In all cases of the CSAFS measurements, had the probe pierced through the protein layer, we would have obtained the I-V response for bare silicon substrate (S8 Fig). But, in this work, the acquired I-V responses for both ferritin and collagen films differed widely from the I-V characteristics for the bare silicon substrate even at high force (about 70 nN), meaning that the I-V response curves were indeed acquired from the protein layer, and not the underlying substrate. We also confirm from the I-V curves as acquired from the heat-treated protein films (S9 Fig) that the I-V curves presented in Figs 5–7 were acquired from protein films that were not denatured, since had they been denatured or the proteins conformationally severely altered due to long storage, the relevant I-V curves would have appeared similar to those presented in S9 Fig, i.e., an I-V curve with a band gap would appear only when the sweep voltage was increased to a much higher value than normally applied, and widened band gap would have been observed.

**Fig 8:**
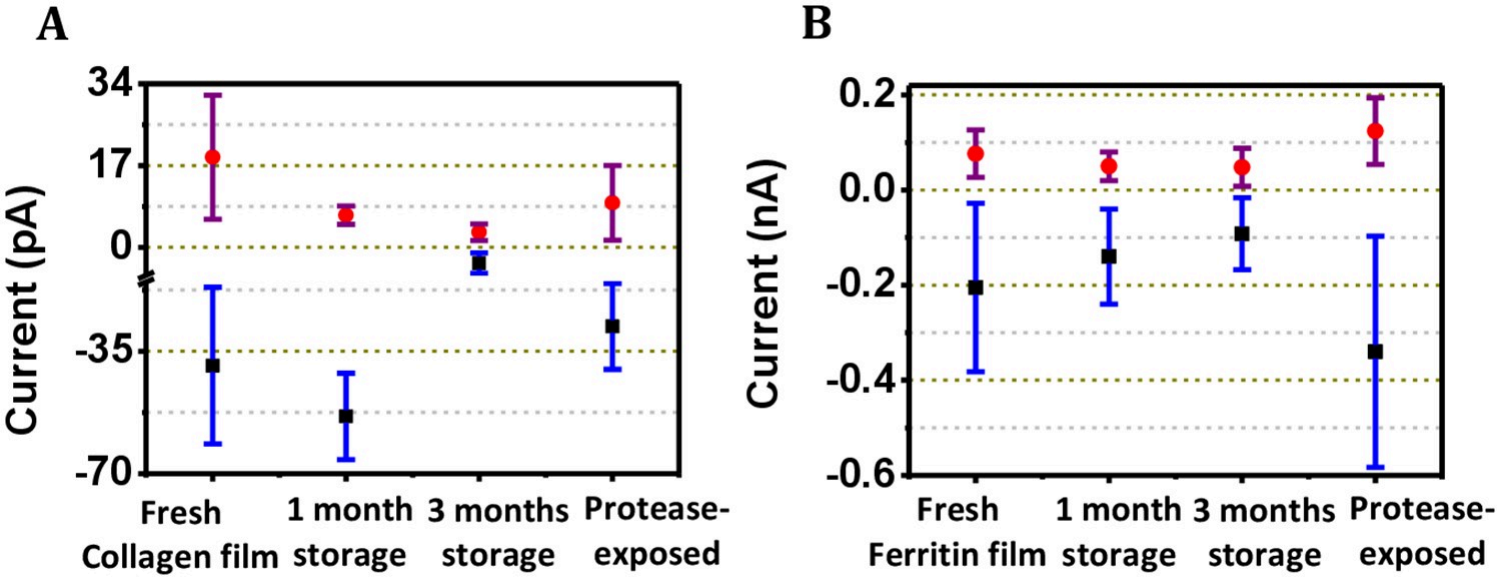
Data variability of the current values for proteins at the different storage conditions. For (A) collagen and (B) ferritin proteins, the variations represented by the purple lines and the blue lines are obtained from the current data points above the zero line and below the zero line, respectively, in the I-V curves.

We determined data variability of the current values measured at high force (71–75 nN) for both collagen and ferritin proteins considering different storage conditions (Fig 8). In case of collagen, when measured at the sweep voltages -4.5 V and 4.5 V, the respective current values were found to be -39.1 ± 22.4 pA and 18.7 ± 12.9 pA (for freshly prepared collagen film); -53.6 ± 12.4 pA and 6.7 ± 1.9 pA (for collagen film stored for one month); -3.3 ± 2.1 pA and 3.2 ± 1.7 pA (for collagen film stored for three months); and -27.9 ± 12.3 pA and 9.3 ±7.8 pA (for protease-exposed collagen film) (Fig 8A). In case of ferritin, the current values when measured at the sweep voltages -2.8 V and 2.8 V, were found to be -0.2 ± 0.18 nA and 0.08 ± 0.05 nA (for freshly prepared ferritin film); -0.14 ± 0.1 nA and 0.05 ± 0.03 nA (for film stored for one month); -0.09 ± 0.07 nA and 0.05 ± 0.04 nA (for film stored for three months); and -0.34 ± 0.24 nA and 0.12 ± 0.07 nA (for protease-exposed ferritin film) (Fig 8B). Although the variation was large in some cases, it should not be due to any instability in the measurement. Rather, the variation could arise from heterogeneity in the protein film structure and consequent variation in the exact location on the protein molecules in the film from where the current data was recorded. Data variation could also arise from the varying state of hydration on the protein film. In order to test if the protein films degraded due to storage over different time periods or for different storage conditions, the significant difference between the current values was obtained for the cases (i) freshly prepared protein film and film stored for one month, (ii) freshly prepared protein film and film stored for three months, (iii) freshly prepared protein film and protease-exposed protein film, by performing a two-tailed t test. The p values for all the mentioned cases were found to be <0.0001 (S2 Table). Therefore, the acquired data is statistically significant and we conclude that the protein films remained largely intact over the tested time periods and storage conditions.

As per the previous reports on the thermal stability of a recombinant ferritin [57] and fully hydrated collagen [58], these proteins remain stable till ~72 °C and ~60 °C, respectively. In case of ferritin, a higher temperature of >80 °C was reported to be responsible for irreversible denaturation of ferritin [22]. As the structural stability of a protein is an important determinant for its proteolytic susceptibility [59], the protease-resistant behavior of the collagen and ferritin proteins as observed in our study indicate that both the proteins are structurally enduring. The most common motif in the amino acid sequence of collagen is Gly-Xaa-Yaa triplets, where Xaa and Yaa can be any amino acid but mostly proline and hydroxyproline. The presence of hydroxyproline residues is thought to be important for the hydrothermal stability of collagen because the hydroxyproline residues can form interchain H-bonds between its hydroxyl group and carbonyl oxygen of an adjacent peptide group [60]. Ferritin, having a protein cage structure, is a highly symmetrical protein with 24 subunits arranged in an octahedral (432) symmetry. The tight packing arrangement of ferritin leads to its high thermal and chemical stability [61]. The structural stability of the collagen and ferritin proteins was further confirmed from the observation of no significant structural change in case of the protease-exposed collagen and ferritin proteins compared to the native proteins as confirmed by their respective CD spectra. Although some degree of cleavage was reported for collagen [53], no cleavage of the ferritin protein by protease could be observed in SDS-PAGE electrophoresis experiments [54]. The electronic structures for both the proteins were also unchanged in presence of the protease from environmental sources and in presence of the deliberately added bacterial proteinase since the UV peak positions for both the proteins remained fixed at their characteristic wavelengths. The structural stability of the two proteins was duly reflected in the functional stability of the proteins as delineated in the CSAFS-derived current-voltage response curves. Both the proteins in film condition were capable of maintaining their natural electron transport capacity even after storage over few months. The previous observations of current values in pA and nA levels for the freshly prepared collagen and ferritin proteins, respectively [27,42], could be repeated in case of both the protein films prepared from either fresh protein solution or stored (environment-exposed) protein solution. This indicates that both collagen and ferritin proteins remained functionally active even under protease exposure and in the stored film state.

## Conclusions

The action of the protease that is present in the ambient environment and of the bacterial protease that is deliberately applied in the collagen and ferritin proteins did not result in any significant change in the secondary structure and the electronic structure of the proteins in comparison to that of their native forms. The protease-exposed collagen and ferritin proteins remained functionally stable for a few months since we found that the electron transport capacity of the protease-exposed proteins was almost comparable with that of the native ones, even over a nine months’ test period. Therefore, we conclude that both the proteins are reasonably stable bioelectronic materials and suitable for potential use in bioelectronics devices.

## Supporting information

S1 Fig AFM images along with cross-section of scratched regions of proteins. (DOC)

S2 Fig CD spectra of protease, heat-treated proteins. (DOC)

S3 Fig Magnified view of UV-visible spectra for environmentally exposed proteins. (DOC)

S4 Fig UV-visible spectra of protease. (DOC)

S5 Fig UV-visible spectra of heat-treated proteins. (DOC)

S6 Fig I-V data of proteins showing on-state at both the voltages. (DOC)

S7 Fig I-V data for 9 months stored proteins. (DOC)

S8 Fig I-V data for silicon. (DOC)

S9 Fig I-V data for heat-treated proteins. (DOC)

S1 Table On-off states in I-V curves of proteins. (DOC)

S2 Table p values for proteins. (DOC)
