# Supplementary material for: How stable are the collagen and ferritin proteins for application in bioelectronics?
Source: PLoS One. 2021 Jan 29;16(1):e0246180. doi: 10.1371/journal.pone.0246180 (PMC7845979; doi:10.1371/journal.pone.0246180)
Supplement: S1 Fig — (DOC) [file pone.0246180.s001.doc]

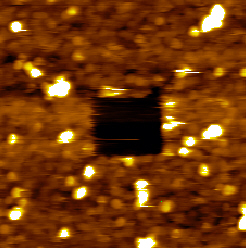


**500 nm**


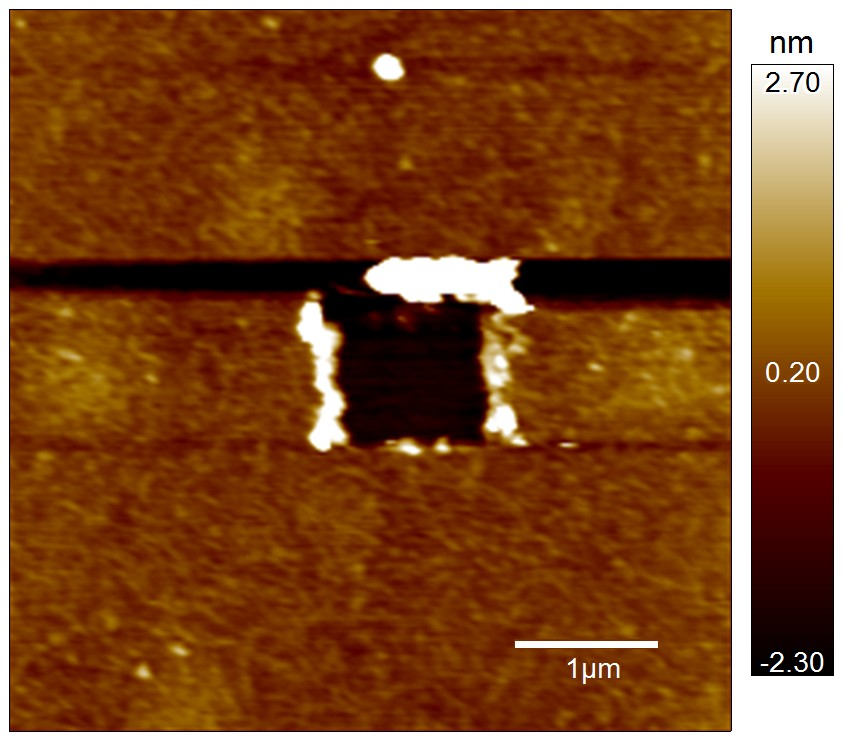

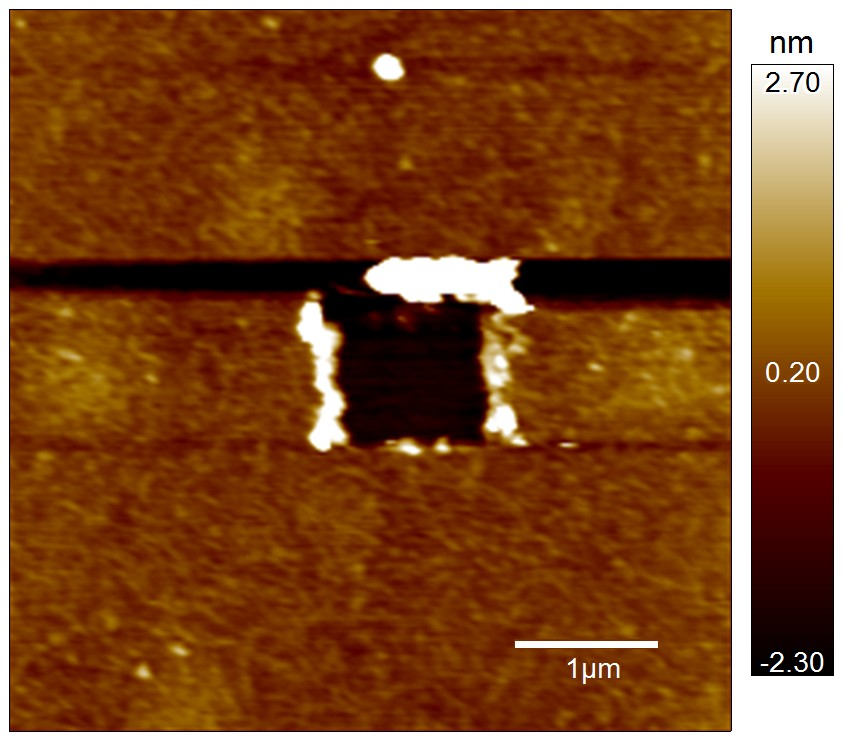


**1 μm**

**A1**

**A2**

**B2**

**B1**

**S1 Fig.** AFM topography images of the scratched regions of (A1) collagen, and (B1) ferritin protein films on silicon substrate. The cross-sections over the scratched regions provide the thickness values of (A2) 1.95 nm for the collagen film and (B2) 9.71 nm for the ferritin film.
