# Supplementary material for: How stable are the collagen and ferritin proteins for application in bioelectronics?
Source: PLoS One. 2021 Jan 29;16(1):e0246180. doi: 10.1371/journal.pone.0246180 (PMC7845979; doi:10.1371/journal.pone.0246180)
Supplement: S2 Fig — (DOC) [file pone.0246180.s002.doc]

**B**

**C**

**A**

**S2 Fig.** Far UV-CD spectra of (A) heat-treated collagen, (B) protease-only case (protease concentration: 8.58 μg/mL), and (C) heat-treated ferritin.
