# Supplementary material for: How stable are the collagen and ferritin proteins for application in bioelectronics?
Source: PLoS One. 2021 Jan 29;16(1):e0246180. doi: 10.1371/journal.pone.0246180 (PMC7845979; doi:10.1371/journal.pone.0246180)
Supplement: S3 Fig — (DOC) [file pone.0246180.s003.doc]

**B**

**A**

**S3 Fig.** Magnified view of UV-visible spectra of (A) collagen and (B) ferritin solution upon environmental exposure at day 1 ( ), day 3 ( ) and day 7 ( ).
