# Supplementary material for: How stable are the collagen and ferritin proteins for application in bioelectronics?
Source: PLoS One. 2021 Jan 29;16(1):e0246180. doi: 10.1371/journal.pone.0246180 (PMC7845979; doi:10.1371/journal.pone.0246180)
Supplement: S4 Fig — (DOC) [file pone.0246180.s004.doc]

**S4 Fig.** UV-visible spectra of protease solution (protease concentration: 8.58 μg/mL).
