# Supplementary material for: How stable are the collagen and ferritin proteins for application in bioelectronics?
Source: PLoS One. 2021 Jan 29;16(1):e0246180. doi: 10.1371/journal.pone.0246180 (PMC7845979; doi:10.1371/journal.pone.0246180)
Supplement: S5 Fig — (DOC) [file pone.0246180.s005.doc]

**A**

**B**

**S5 Fig.** UV-Vis spectra of (A) collagen and heat-treated collagen, (B) ferritin and heat-treated ferritin.
