# Supplementary material for: How stable are the collagen and ferritin proteins for application in bioelectronics?
Source: PLoS One. 2021 Jan 29;16(1):e0246180. doi: 10.1371/journal.pone.0246180 (PMC7845979; doi:10.1371/journal.pone.0246180)
Supplement: S6 Fig — (DOC) [file pone.0246180.s006.doc]

**A**

**B**

**S6 Fig.** The I-V curves showing ‘on-state’ at both the positive and negative voltage sides using extrapolated lines touching x-axis for (A) collagen, (B) ferritin films stored for different time intervals.
