# Supplementary material for: How stable are the collagen and ferritin proteins for application in bioelectronics?
Source: PLoS One. 2021 Jan 29;16(1):e0246180. doi: 10.1371/journal.pone.0246180 (PMC7845979; doi:10.1371/journal.pone.0246180)
Supplement: S7 Fig — (DOC) [file pone.0246180.s007.doc]

**A**

**80**

**-160**

**-9**

**9**

**V**

**I**

**0**

**0**

**B**

**S7 Fig.** Current (I) vs. voltage (V) curves for (A) collagen and (B) ferritin films that are freshly prepared ( ), and films stored for nine months ( ) at 71-75 nN force applied. In the inset of panel A, I-V curve for stored collagen film was shown at ± 9 V sweep range.
