# Supplementary material for: How stable are the collagen and ferritin proteins for application in bioelectronics?
Source: PLoS One. 2021 Jan 29;16(1):e0246180. doi: 10.1371/journal.pone.0246180 (PMC7845979; doi:10.1371/journal.pone.0246180)
Supplement: S9 Fig — (DOC) [file pone.0246180.s009.doc]

**B**

**10**

**-10**

**-3**

**3**

**V**

**I**

**0**

**0**

**A**

**S9 Fig.** I-V curves from (A) heat-treated ferritin film (in the inset, the region within -3V to +3V is shown) and (B) heat-treated collagen film, on silicon surface at 71-75 nN applied force.
