# Supplementary material for: How stable are the collagen and ferritin proteins for application in bioelectronics?
Source: PLoS One. 2021 Jan 29;16(1):e0246180. doi: 10.1371/journal.pone.0246180 (PMC7845979; doi:10.1371/journal.pone.0246180)
Supplement: S1 Table — (DOC) [file pone.0246180.s010.doc]

**S1 Table**: The on-off states measured from I-V curves of proteins.

| **On-off states in collagen I-V curve** | | | |
| --- | --- | --- | --- |
|  | Off-state | On-state at positive voltage | On-state at negative voltage |
| Freshly prepared | -3.9 volt to +4.36 volt | > 4.36 volt | < -3.9 volt |
| After one month | -3.44 volt to +4.36 volt | >4.36 volt | < -3.44 volt |
| After 3 month | -4.54 volt to +4.71 volt | > 4.71 volt | < -4.54 volt |
| **On-off states in ferritin I-V curve** | | | |
|  | Off-state | On-state at positive voltage | On-state at negative voltage |
| Freshly prepared | -1.34 volt to +0.98 volt | > 0.98 volt | < -1.34 volt |
| After one month | -1.34 volt to +1.25 volt | >1.25 volt | < -1.34 volt |
| After 3 month | -1.42 volt to +1.46 volt | > 1.46 volt | < -1.42 volt |
