# Supplementary material for: How stable are the collagen and ferritin proteins for application in bioelectronics?
Source: PLoS One. 2021 Jan 29;16(1):e0246180. doi: 10.1371/journal.pone.0246180 (PMC7845979; doi:10.1371/journal.pone.0246180)
Supplement: S2 Table — (DOC) [file pone.0246180.s011.doc]

**S2Table.** The p value calculation for both collagen and ferritin proteins for different storage conditions.

| **Type of Protein** | **Sweep voltage at which current values measured** | **Comparison done between** | **p value** |
| --- | --- | --- | --- |
| Collagen | -4.5 V and 4.5 V | Freshly prepared protein film and film stored for 1 month | < 0.0001 |
|  |  | Freshly prepared protein film and film stored for 3 months | < 0.0001 |
|  |  | Freshly prepared protein film and protease-exposed protein film | < 0.0001 |
| Ferritin | -2.8 V and 2.8 V | Freshly prepared protein film and film stored for 1 month | < 0.0001 |
|  |  | Freshly prepared protein film and film stored for 3 months | < 0.0001 |
|  |  | Freshly prepared protein film and protease-exposed protein film | < 0.0001 |
